# Rare case of a bronchogenic cyst at the distal esophagus

**DOI:** 10.1016/j.igie.2025.08.004

**Published:** 2025-08-22

**Authors:** Brian Xu, Saurabh Chawla, Gabriela Oprea-Ilies, Emad Qayed

**Affiliations:** 1 Department of Internal Medicine, Morehouse School of Medicine, Atlanta, Georgia, USA; 2 Department of Gastroenterology, Emory University School of Medicine, Atlanta, Georgia, USA; 3 Department of Pathology, Emory University School of Medicine, Atlanta, Georgia, USA

Bronchogenic cysts are congenital lesions resulting from abnormal budding of the foregut during embryological development. Locations can be found throughout the mediastinum, particularly in the bronchus or within the lung parenchyma.^1^ Involvement of the esophagus is uncommon, and they are often found incidentally on imaging.^1^^,^^2^ Here, we present a case of an incidental bronchogenic cyst within the distal esophagus found on chest computed tomography (CT).

A 79-year-old woman with a medical history of chronic obstructive pulmonary disease presented to our pulmonary clinic for worsening shortness of breath. A chest CT was conducted, and an incidental 1.7-cm soft-tissue lesion (Fig. 1) was identified at the distal esophagus. At her primary care follow-up, she denied dysphagia, odynophagia, or chest pain. However, given the incidental findings, a barium swallow was ordered, and referral was placed to gastroenterology for upper endoscopy evaluation. Barium swallow revealed a round smooth filling defect at the posterolateral distal esophagus wall without obstruction. Upper endoscopy identified a 2-cm subepithelial lesion (Fig. 2) 31 cm from the incisors. Endoscopic ultrasound (EUS) identified a large (18- × 12-mm), hypoechoic, and mildly heterogeneous mass, likely originating from the submucosa (Fig. 3). This mass was mildly deformable upon pressure. Fine-needle aspiration (FNA) was performed with cytology showing bronchial cells with conspicuous cilia, consistent with a diagnosis of a bronchogenic cyst (Fig. 4). At follow-up, given her advanced age and lack of symptoms, the patient opted for clinical monitoring and no further workup.

**Figure 1:**
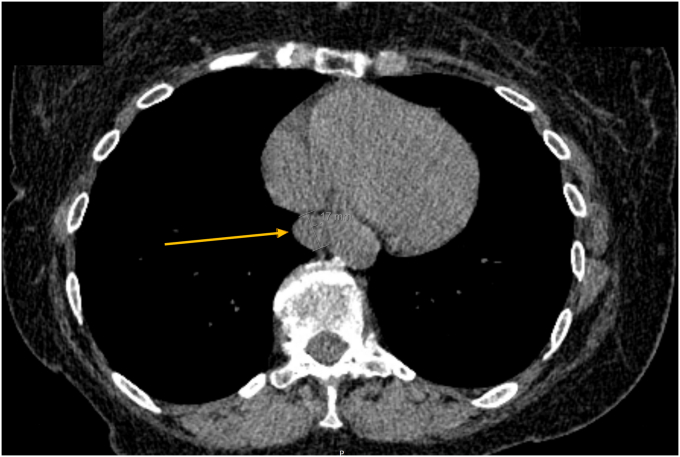
Noncontrast computed tomography of the chest showing a round soft-tissue lesion along the distal esophagus with a mean diameter of 1.7 cm (*yellow arrow*).

**Figure 2:**
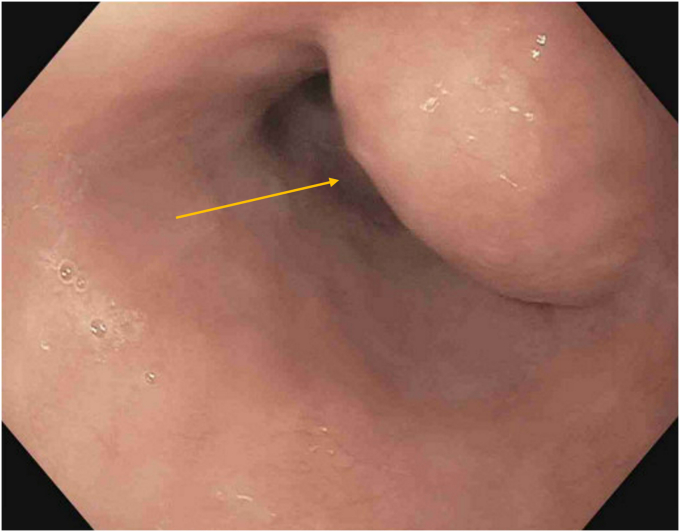
Upper endoscopy showing a 2-cm subepithelial lesion (*yellow arrow*) in the distal esophagus at 31 cm from the incisors.

**Figure 3:**
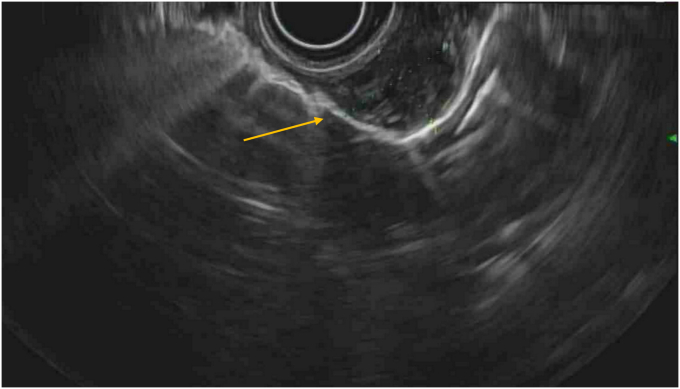
Endoscopic ultrasound image showing an 18- × 12-mm hypoechoic mildly heterogenous mass likely arising from the submucosa (*yellow arrow*).

**Figure 4:**
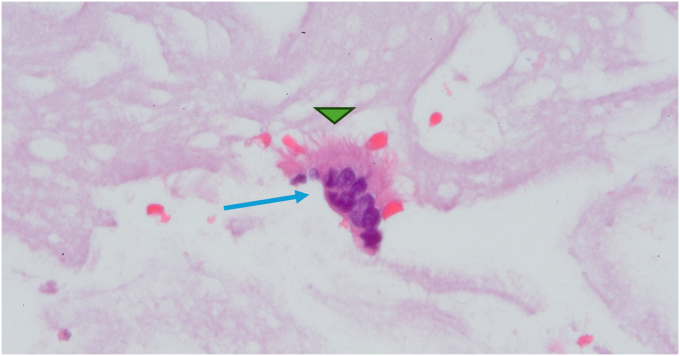
Hematoxylin and eosin staining (original magnification ×60) of the fine-needle aspirate sample showing bronchial cell clusters (*blue arrow*) and mucin with conspicuous cilia (*green arrowhead*).

Esophageal bronchogenic cysts are rare congenital anomalies that are often asymptomatic and typically discovered incidentally during imaging studies.^2^^,^^3^ Further evaluation with an EUS is required to better characterize the lesions, especially aiding in differentiation between cystic versus solid masses. However, the EUS appearance of bronchogenic cyst can vary from hypoechoic to dense hyperechoic debris.^2^ In our case, EUS demonstrated an hypoechoic mass requiring FNA, with cytology showing respiratory cells of an enteric origin. In regard to overall management, surgical resection is often recommended even for asymptomatic cases because of a risk of future adverse events such as infections, rupture, and potential for malignant transformation.^4^ However, there are no clear guidelines for the treatment of asymptomatic esophageal bronchogenic cysts, and that should be individualized on a case-by-case basis.^5^ Taking into consideration our patient’s age of 79 years, lack of symptoms, and benign cytology, it was reasonable to pursue conservative management.

## Patient consent

Informed consent was obtained from the patient for the publication of their information and imaging.

## Disclosure

All authors disclosed no financial relationships.
